# Aggressive breast fibromatosis following augmentation mastoplasty: a series of case reports

**DOI:** 10.3332/ecancer.2018.833

**Published:** 2018-05-11

**Authors:** Rafael Delgado Morales, Armando Gil Mendoza, Carmen Luces, Efren Bolívar Abreu, Gabriel Romero, Gabriel Pérez, Leonardo Russo

**Affiliations:** 1 Department of Digestive Pathology, Soft Tissue Tumors and Melanoma, Instituto de Oncología Luis Razetti (IOLR), Caracas, 1010, Venezuela; 2 Breast Clinic, Carrera 11 68-36, Bogotá, Colombia; 3 Department of Mammary Pathology, Instituto de Oncología Luis Razetti (IOLR), Caracas, 1010, Venezuela

**Keywords:** aggressive fibromatosis, augmentation mastoplasty

## Abstract

Aggressive fibromatosis comprises connective tissue tumours that represent 0.03% of all bodily neoplasms, occurring more often in the abdominal wall, mesentery, and extremities; its location in the breast constitutes a very infrequent type of lesion. Its pathogenesis is diverse and its relationship with augmentation mastoplasty is still unclear. Four cases of aggressive breast fibromatosis following augmentation mastoplasty are reported in this article.

## Introduction

Aggressive fibromatosis comprises connective tissue tumours that represent 0.03% of all bodily neoplasms, occurring more frequently in the abdominal wall, mesentery and extremities [1]. Aggressive fibromatosis of the breast constitutes a very infrequent type of lesion, representing only 0.2% of all breast tumours [2]. The risk of this disease appearing after the insertion of breast implants is not clear, due to a lack of research because of its infrequent appearance [3].

## Case reports

A review of the database of medical histories from the Dr. Luis Razetti Institute of Oncology from the years 2000 to 2017 was conducted; 81 patients with a diagnosis of aggressive fibromatosis were found, of whom only five (6.17%) corresponded to lesions located in the breast. All of the patients were found to be females, and four had a history of augmentation mastoplasty. Cases with a history of mamoplasty will be described in Table 1.

## Case report 1

The first patient, 21 years of age, presented with a progressive, nonpainful increase in volume of the left breast; the patient had augmentation mastoplasty 19 months ago, when silicone implants were inserted. Physical examination showed asymmetry in the juncture of the upper quadrants, and a hard, nonmob disease-free interval (DFI) tumour of 6 × 6 cm^2^ was felt. Breast ultrasound showed a heterogeneous, hypoechoic, solid tumour of 6 × 2 × 4 cm^3^ on the surface of the major pectoral; no lesion was observed on the mammogram. Chest tomography with intravenous contrast produced a collective heterogeneous image, and on the magnetic resonance image of the breast, an irregular image is shown on the anterior surface of the left major pectoral, isointense in T1 and hyperintense in T2; the administration of contrast shows a progressive impregnation curve.

The core needle biopsy shows a fusocellular tumour consonant with extra-abdominal fibromatosis, and the immunohistochemistry showed positive immunoreactivity for vimentin and negative for desmin, CD34 and S100 protein. With this diagnosis, a wide local excision (WLE) of the tumour was conducted, which included the anterior segment of the second and third ribs, in addition to the removal of the prosthesis. The reconstruction of the thoracic wall was performed with polypropylene mesh. The definitive biopsy revealed a tumour compatible with aggressive fibromatosis, microscopically positive, with superior and medial margins, for which the patient received adjuvant radiotherapy (Rt).

The patient experienced a local relapse 34 months after surgery, for which a WLE was performed with excision edges; the result was negative for disease and patient currently has a DFI of 22 months.

## Case report 2

The second patient, 31 years old, presented with a tumour of the left breast of 7 months of progression; 6 months earlier the patient had undergone augmentation mastoplasty in which silicone implants were inserted. At the initial physical examination a hard, nonmobDFI tumour of 5.5 cm was felt in the upper inner quadrant. Lesions were not observed in the mammogram; the tomography of the thorax with intravenous contrast showed the rear bilateral pectoral prosthesis with an undulated surface; in the left fore-pectoral there was a 7 × 4.5 cm^2^ lesion of mixed density. On the breast magnetic resonance imaging (MRI), a solid, heterogeneous mass was observed in the left breast.

The core needle biopsy revealed a desmoid tumour. With this diagnosis, a WLE of the tumour and removal of the prosthesis was performed. The definitive biopsy revealed a tumour consistent with aggressive extra-abdominal fibromatosis, showing a pattern of infiltrative growth, with edges negative for disease; patient did not receive adjuvant treatment. After 36 months of follow-up care the patient is free of locoregional and distant disease.

## Case report 3

The third patient, 33 years old, presented with a tumour of the left breast of 5 months of progression; 15 months earlier the patient underwent augmentation mastoplasty in which silicone implants were inserted. At the physical exam a hard, nonmobDFI tumour of 5 × 5 cm^2^ is felt in the upper external quadrant of the breast. Lesions are not observed in the mammogram; the tomography with intravenous contrast of the thorax showed the rear bilateral pectoral prosthesis; in the area of the left fore-pectoral there is a solid 6 × 4.5 cm^2^ lesion; the breast MRI showed a solid heterogeneous mass.

**Figure 3.**
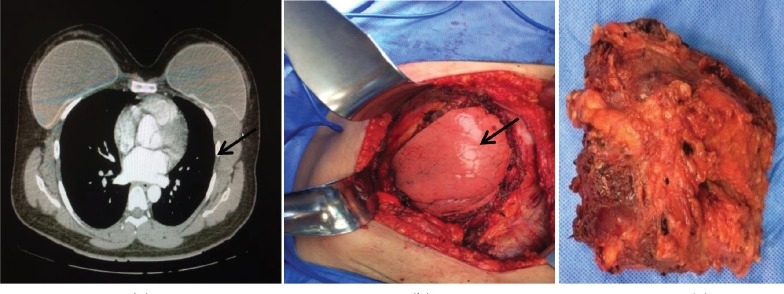
Third case. (a) Patient with fibromatosis of the left breast in the CT of the thorax: solid lesion adjacent to the mammary prosthesis. (b) At the moment of the surgical resection: WLE area, observing the lung. (c) Surgical specimen (WLE).

The core needle ultrasound guided biopsy revealed a desmoid tumour, and this was confirmed through immunohistochemistry. With this diagnosis, a WLE of the tumour was conducted, which included the segment of the second and third ribs, as well as the removal of the prosthesis. The reconstruction of the thoracic wall was performed with polypropylene mesh (Figure 3). The definitive biopsy revealed a tumour consistent with aggressive fibromatosis, with edges negative for disease; the patient did not receive adjuvancy. After 24 months of follow-up care, the patient is free of locoregional and distance disease.

## Case report 4

**Figure 4.**
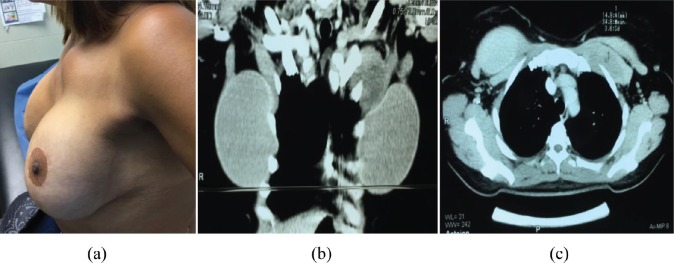
Fourth case. (a) Patient with fibromatosis of left breast at initial physical exam: tumour of left breast. (b) CT of the thorax, coronal incision. (c) CT of the thorax, axial incision: heterogeneous lesion retropectorally located.

The fourth patient was 42 years old, with illness onset 10 months prior to the first consult when the patient presented with a tumour of the left breast; 20 months earlier the patient underwent augmentation mastoplasty. The lesion was felt at the union of the upper quadrants, 6 cm from the nipple-areolar complex, measuring 5 × 5 cm^2^, hard, nonmobDFI, extending to the subclavicular area. Thorax tomography showed a heterogeneous lesion located retropectorally adjacent to the rear plane of the prosthesis and extending along the medial clavicular line from the first rib to the third rib; a study of the mammogram revealed no lesions (Figure 4). The core needle biopsy revealed aggressive fibromatosis, and this was confirmed through immunohistochemistry.

A WLE of the tumour was conducted, which included the segment of the second and third ribs, as well as the removal of the prosthesis. The reconstruction of the thoracic wall was performed with polypropylene mesh (Figure 3). The definitive biopsy revealed a tumour consistent with aggressive fibromatosis, with edges negative for disease; patient did not receive adjuvant treatment. After 18 months of follow-up care, the patient is free of locoregional and distance disease.

## Discussion

Aggressive fibromatosis was initially called desmoid tumour, a word which derives from the Greek ‘Desmos’ (which means band or similar to the tendon); Muller described it in this way for the first time in 1838, when he described tumours with a similar consistency to tendons. The term fibromatosis was originally introduced by Stout, to define a group of clinical conditions (plantar fibromatosis, juvenDFI, deep, lipofibromatosis, multiple hyaline fibromatosis, scarring fibromatosis, etc.) that have the following features in common: proliferation of well-differentiated fibroblasts, infiltrative growth pattern, the presence of a variable amount of collagen between the proliferating cells, absence of cytologic features of malignancy, absence or very low mitotic activity and aggressive clinical behaviour characterised by a high rate of local relapse with low capacity to metastasize [3, 4].

Macroscopically these lesions are often voluminous, firm, whitish and with poorly defined edges, originating almost entirely from the muscle fascia. Microscopically the majority of cells have intermediate characteristics between fibroblasts and smooth muscle (myofibroblast) cells, in addition to the characteristics already referred to in Stout’s criteria. Fibromatosis cells have a focal and often erratic immunoreactivity to alpha-smooth muscle actin, vimentin, desmin, calponin, and oestrogen receptors [5]. There is still little known regarding the etiopathogenesis of aggressive fibromatosis with genetic predisposition (patient with familial polyposis adenomatous of the colon); traumas, hormonal effects (oestrogen), among many other factors are strongly associated with their development [6].

Precise records about the incidence and prevalence of aggressive breast fibromatosis do not exist in Venezuela; according to records from other countries it is estimated that this illness represents only 0.2% of all breast tumours [7, 8]. The appearance in relation to the placement of breast implants is not frequent, and only 25 cases have been reported in the English-speaking literature, 3 of those cases lack detaDFId descriptions of the disease. Of those 22 cases with complete descriptions, 16 were with silicone implants and 6 were with saline; the average time between implant placement and the onset of the disease was 3 years; 75% were treated with WLEs without adjuvant treatment and in most cases the implant was removed. The rate of recurrence in this group of patients was 25%, with an average time of appearance of 34.3 months (Table 2).

The clinical presentation of breast fibromatosis is characterised by a non-painful breast tumour, no trophic changes of skin, firm and nonmobDFI, since many of these injuries originate outside of the mammary parenchyma in the thoracic wall; because of this, it is common that a lesion is not seen on the mammogram; however, when observed, it presents as spiculated lesions, with poorly defined and irregular edges without calcifications. MRI is a primary tool in the evaluation of patients in whom this illness is suspected, because of the above mentioned clinical feature [3].

Our study is comprised of young women (average age of 31.5 years); the type of prosthesis utilised was silicone, and surgical history of augmentation mammoplasty had occurred 2 years prior to the appearance of the tumour, data which is consistent with the total number of cases reported to date in the literature.

The recommended treatment for patients with aggressive fibromatosis following augmentation mammoplasty is WLE, in addition to the removal of the prosthesis, the immediate or later replacement of which is described in some cases [1, 3, 7]. Although the previous behaviour is based on a study with a low level of evidence, the biological nature of fibromatosis WLE plus removal of the adjacent prosthesis is the oncologically accepted treatment; controversies arise in making immediate or deferred replacement after optimal treatment; in making this decision, aesthetic, emotional and cost-benefit aspects should be analysed. Taking data from our institution, deferred replacement could be the best option, since up to 25% of patients could receive adjuvant Rt, altering the postoperative evolution of those patients who get reconstruction in the first place [23].

Radical treatment with prosthesis removal was performed on all of the patients in the study, requiring multiple costal resections in 3 of the cases. Adjuvant treatment with Rt was recommended for only one patient, due to the definitive biopsy report of a resection with microscopic disease on the edges of the resection; this patient presented with local recurrence of the disease, was treated successfully with WLE, and up to the date of publication, all of the patients are alive without signs of recurrence of the disease.

Adjuvant treatment in patients with aggressive breast fibromatosis is not clear; each case should be considered individually with multidisciplinary discussions. Radiation therapy, hormone replacement therapy, immunotherapy and chemotherapy are some of the options that we have; however, there are no pharmacological or radiotherapeutic studies that support any of them.

## Conclusions

Aggressive fibromatosis of the breast is a rare disease that represents 0.2% of all breast tumours; there is currently little known about the etiopathogenesis of aggressive fibromatosis and its relationship with breast augmentation is not clear with few cases reported. The diagnosis is made through preoperative biopsy and imaging studies (mammography, MRI and CT) play an essential role in planning the definitive treatment. WLE with resection edges negative for disease is the main therapeutic strategy.

## Figures and Tables

**Figure 1.**
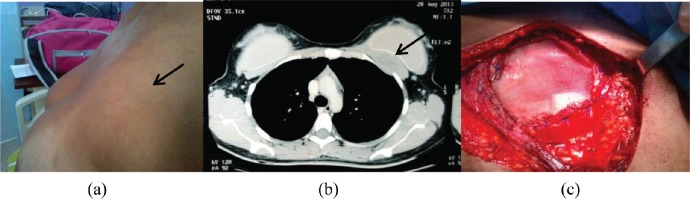
First case. (a) Patient with breast fibromatosis at initial physical exam: asymmetry at the union of the superior quadrants. (b) Computed tomography (CT) of the thorax: area of tumour adjacent to the prosthesis is shown. (c) At the moment of the surgical resection: area of WLE reconstructed with polypropylene mesh.

**Figure 2.**
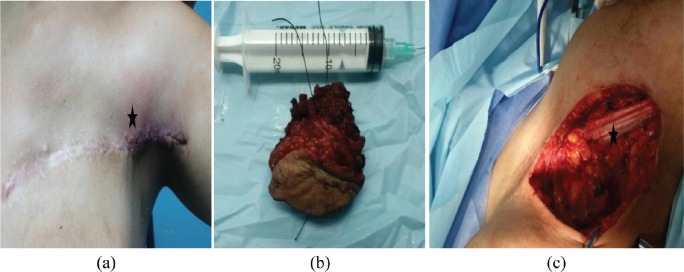
Patient with local relapse. (a) Physical examination where a palpable nodule of 3 × 3cm^2^ on the outer third of scar is observed. (b) Surgical specimen. (c) Tumour bed.

**Table 1.** Features of the cases registered in the study.

Case	Age (years)	Biomaterial	Tumour size	Interva (months)	Treatment	Results (months)
1	21 (Figure 1, 2)	Silicone	6×6 cm^2^	19	WLE + removal of prosthesis + Rt	Recurrence at 34 months. T to WLE with DFI 22 months
2	31	Silicone	5×5 cm^2^	16	WLE + removal of prosthesis	DFI 36 months
3	33 (Figure 3)	Silicone	6×6 cm^2^	15	WLE + removal of prosthesis	DFI 24 months
4	42 (Figure 4)	Silicone	5×4 cm^2^	20	WLE + removal of prosthesis	DFI 18 months

WLE: Wide local excision; NR: Not reported; Rt: Radiotherapy; DFI: Disease-free interval

**Table 2.** Characteristics of reported cases of aggressive fibromatosis associated with breast implants.

Author	Age	Biomaterial	Tumour size	Interval (years)	Treatment	Results (months)
Jewitt and Mead [8]	54	Saline	3 cm	2	WLE + removal of prosthesis	DFI: 8
Rosen and Ernsbeerger [9]	35	Saline	NR	NR	Excisional Bx + removal of prosthesis	1st recurrence 7 months and 2nd 12 months, DFI:12
Schuh and Radford [10]	41	Silicone	6.5 cm	2	WLE + removal of prosthesis	DFI: 36
Schiller *et al* [11]	66	Silicone	13 cm	NR	WLE + removal of prosthesis	NR
Dale *et al* [12]	65	Silicone	13 cm	7	WLE + removal of prosthesis	NR
Crestinu [13]	NR	Silicone	NR	2	WLE + replacement of prosthesis	DFI: 90
Aaron *et al* [14]	43	Saline	NR	6	WLE + removal of prosthesis + Rt (45 cGy)	Recurrence at 96 months
Vandeweyer and Deraemaecker [15]	45	Silicone	3 cm	3	Excisional Bx + QT + WLE + Removal of prosthesis + HT	DFI: 24
Abraham *et al* [16]	55	Silicone	6 cm	NR	WLE + replacement of prosthesis + RT	NR
Khanfir *et al* [17]	52	Saline	8 cm	1.6	WLE + removal of prosthesis	Recurrence 8
Jandali *et al* [18]	24	Silicone	6 cm	9	WLE + removal of prosthesis	Recurrence 36
Gandolfo *et al* [19]	22	Silicone	16 cm	2	WLE + removal of prosthesis	NR
Jamshed *et al* [20]	30	Saline	5.6 cm	3	WLE + removal of prosthesis	DFI: 24
Neuman *et al* [21]	64 NR 37 28 38 29	Silicone Silicone Silicone Saline Silicone Silicone	6.7 cm 4.5 cm 3.3 cm 11 cm 12 cm 7.4 cm	1.8 2 2.5 2 2 2	WLE + removal of prosthesis	DFI: 40 DFI: 48 DFI: 42 DFI: 36 Recurrence 24 DFI: 92
Chummun *et al* [7]	34	Silicone	9 cm	2	Excisional Bx + replacement of prosthesis + Qt	DFI: 55
Matrai *et al* [1]	34	Silicone	4 cm	2	Excisional Bx + removal of prosthesis + HT	DFI: 28
Brown *et al* [3]	39	Silicone	5 cm	7	WLE + removal of prosthesis	DFI: 24

WLE: Wide Local Excision; NR: Not Reported; Qt: Chemotherapy; HT: Hormone Replacement Therapy; DFI: Disease-Free Interval.
